# Snapshot on Cell-to-Cell Communication Nanotubes: From Bacteria to Humans

**DOI:** 10.3390/biology15060513

**Published:** 2026-03-23

**Authors:** Loredana Moro

**Affiliations:** Human Oncology and Pathogenesis Program, Memorial Sloan Kettering Cancer Center, New York, NY 10065, USA; morol@mskcc.org

**Keywords:** bacterial nanotubes, septal pores, plasmodesmata, cytonemes, tunneling nanotubes, dendritic nanotubes, mitochondrial transfer

## Abstract

From bacteria to higher vertebrates, cells have developed different systems to communicate with each other and transmit specific signals in a dynamic interplay with neighborhood cells. This review focuses on cell-to-cell communication mediated by nanotubes, membrane protrusions present during evolution from bacteria to higher plants and animals. We describe the basic structure of nanotubes in different organisms and cell types and their functions, which cover transfer of signaling molecules, ions, organelles and pathogens in a cell- and context-dependent manner, thereby promoting cell survival, tissue development, response to stress, pathogens’ spreading and drug resistance. We also provide an overview of recent studies that are broadening our understanding of the role of these conduits in the pathogenesis of high-incidence diseases in humans, such as cancer and neurodegeneration.

## Simple Summary

Just like we communicate with other human beings or with a cat, a dog, or other living organisms through different types of language, including verbal language, body language and eye contact, cells have developed several mechanisms to communicate with each other and transmit various types of information. The focus of this review is a specific form of intercellular communication, i.e., nanotubes, which are present in simple unicellular organisms, like bacteria, up to more complicated multicellular organisms, such as higher vertebrates. Research on nanotubes has gained momentum in recent years, owing to the discovery that this communication system is involved in highly prevalent diseases in humans, including neurodegeneration and cancer. This review uncovers the structure and function of nanotubes in different kingdoms of life, from prokaryotic cells to human cells, and includes recent research pointing to the role of these conduits in cancer cell immune evasion and survival, in viral infection, and in neurodegenerative diseases.

## 1. Introduction

Cell-to-cell communication can be defined as the way cells interact to respond to stress, maintain homeostasis, differentiate, and give rise to tissues and organs. Cell-to-cell communications are essential in multicellular organisms, being pivotal in regulating development and homeostasis. In recent years, long-range cell communications mediated by plasma membrane protrusions have emerged as a significant intercellular signaling route both in prokaryotes and eukaryotes. These tubular conduits between cells were first identified in multicellular organisms, with cytoplasmic connections observed in plants a century ago and termed “plasmodesmata” [1]. Decades later, plasmodesmata were visualized in green algae as pores lined by a plasma membrane joining the cytoplasm of neighboring cells [2]. Notably, some unicellular eukaryotes, such as green algae, tend to form multicellular aggregates that are thought to represent a crucial evolutionary transition from unicellular organisms to multicellular forms of life (higher plants in the plant kingdom). In this context, the formation of plasmodesmata in algae can be seen as one of the first evolutionary milestones to establish the cell-to-cell contacts essential for multicellular life [2].

In 1961, membrane protrusions were observed in developing sea urchin larvae [3], but only in 1999 were cell-to-cell membrane nanotubes described in more detail and their function as transport system started to be characterized in invertebrates (*Drosophila melanogaster*) and vertebrates (*Mus musculus*) [4]. These intercellular nanotubes have been termed differently, depending on details of their structure and/or tissue localization and specific signaling molecules transferred. Surprisingly, it was not until 2011 that analogous nanotubular conduits exchanging intracellular content were identified among bacterial cells of the same and different species [5], indicating that nanotubes represent an ancient communication system shared by prokaryotes and eukaryotes.

This review delves into the structure and function of intercellular nanotubes in bacteria, fungi, algae, higher plants, invertebrates and vertebrate organisms, including *Homo sapiens*. Furthermore, this review summarizes and discusses emerging research on the role of these cell-to-cell communication conduits in physiological and pathological conditions and their implications in human diseases, with a focus on viral infection, neurodegeneration and cancer.

## 2. Overview of Nanotubes’ Structure

Nanotubes are membranous cell projections that interconnect neighboring cells. Their diameter is different in different organisms, with a minimum diameter of 2 nm for plants’ plasmodesmata and a maximum diameter of 1 μm in thick TNTs in animal cells (Table 1 and References therein). Similarly, the nanotube length is extremely variable, with a minimum length of 100 nm in bacteria up to connecting an entire multicellular organism in plants (symplast). In addition, while nanotubes are transient structures in bacteria and animals, intercellular nanotubes in plants, green algae and fungi are permanent structures. The permanent nature of these structures in green algae represent an initial milestone for establishing the intercellular contacts essential for multicellular organisms [2]. Septal pores in fungi, and plasmodesmata in plants, represent an essential intercellular connectivity system that allows transport of nutrients and signals through the whole organism [6,7]. These intercellular bridges are surrounded by a cell wall both in fungi (chitin, [8]) and plants (pectin > cellulose, [9]). This characteristic, together with the functional significance of the nanotubular connections in the context of the entire organism, could explain their permanent nature in these multicellular organisms.

## 3. Nanotunnels/Nanotubes in Prokaryotic Cells

Geochemical research provides evidence that bacteria are the earliest form of life and originated more than 3.5 billion years ago [31,32]. Bacteria, still one of the most prevalent forms of life on Earth, have adopted several strategies to adapt to and thrive into the most disparate environmental conditions. The formation of transient tubular membranous structures to connect neighboring cells is among these “adaptation” strategies. The first study of the formation of intercellular tunnels in bacteria was published in 2011 [5]. These structures, termed nanotubes in *Bacillus subtilis* [5] and *Escherichia coli* [10], nanopods in hyperthermophilic archae of the genus *Thermococcus* [33], and outer membrane tubes in *Myxococcus xanthus* [34], are composed of cytoplasmic membranes or outer membranes, depending on the species. *Bacillus subtilis* is the model organism where bacterial nanotunnels have been best characterized [5]. Electron microscopy revealed that tubular extensions (nanotubes) connecting neighboring *B. subtilis* cells have a typical inner diameter of 30–130 nm and can transfer metabolites (such as amino acids), toxins and proteins conferring transient antibiotic resistance, as well as nonconjugative plasmids conferring hereditary characteristics to recipient cells [5,10,11]. Ymdb, an enzyme that hydrolyzes cyclic nucleotides such as cyclic AMP (cAMP), localizes to nanotubes in *B. subtilis* and is necessary for their formation [35], together with proteins of the CORE flagellar export apparatus [36]. YmdB genetic ablation causes a 95% reduction in membrane protrusions and a parallel 25-fold decrease in the number of antibiotic-resistant colonies [35], supporting the biological significance of bacterial membrane nanotunnels as intercellular communication highways.

The genetic ablation of the enzymes involved in the biosynthesis of the amino acids histidine and tryptophan leads to rapid formation of nanotubes and bidirectional cytoplasmic exchange among neighborhood cells, whereas supplementation of tryptophan and histidine in the growth media abolishes the formation of membranous protrusions [10], indicating that nutrient availability is a triggering factor for the formation of nanotubes in bacteria. Notably, bacterial nanotubes can be formed not only among cells of the same species but also between different species, such as between *B. subtilis* and *Staphylococcus aureus*, and *B. subtilis* and *Escherichia coli* [5]. Intriguingly, nanotubes can also directly connect bacteria with a eukaryotic host, as demonstrated for enteropathogenic *E. coli*, a human-specific pathogen that can cause acute intestinal diseases. Enteropathogenic bacteria use this connection strategy to extract nutrients from the host cells and replicate. The formation of these bacteria–eukaryotic communication tunnels is dependent on the expression of functional flagellar proteins of the CORE apparatus [37]. Studies in different bacteria species confirmed that the CORE-containing nanotube is a ubiquitous organelle that promotes intercellular traffic of molecules across the bacterial kingdom [36].

It should be mentioned that, though most of the literature so far indicates that bacterial nanotubes facilitate intercellular trade of nutrients, cytoplasmic proteins, and plasmids, a study using *B. subtilis* shows that nanotubes are death-related structures involved in cell disintegration and, therefore, would not be involved in active cytoplasmic exchange among cells [38]. Despite the contradictory results for *B. subtilis*, more recent evidence has extended the presence and functional activity of bacterial nanotubes to marine cyanobacteria *Synechococcus* and *Prochlorococcus*, two relevant species for the Earth’s ecosystem, as they are responsible for one-fourth of the primary production in the ocean [39]. Marine cyanobacteria were shown to exchange cytoplasmic material with each other between neighboring and distant cells [40]. In addition, the authors excluded the possibility that nanotubes in cyanobacteria were produced by dying cells, as previously reported in *B. subtilis* [38,40]. Regarding the components of the nanotubes in cyanobacteria, a BLASTP search detected no hits for components of the CORE system in the cyanobacterial genomes, suggesting that cyanobacteria may use an alternative system to build nanotubes [40]. In addition to cyanobacteria, the presence of nanotubes has also been observed in the heterotrophic marine bacteria *Pseudoalteromonas* sp. TW7 and *Alteromonas* sp. ALTSIO [12], though active cytoplasmic traffic has not been studied in these bacteria. In heterotrophic marine bacteria, individual bacterial nanotubes measured 50–160 nm in width and displayed a length of 100–600 nm between connected cells [12]. Using marine phototroph–heterotroph interaction systems, it has been demonstrated that although cyanobacteria are specialized for performing photosynthesis and carbon fixation, they rely on heterotrophic bacteria to breakdown the leaked organic matter into its basic inorganic forms, suggesting a mutualistic, non-competitive interaction that would benefit both organisms by allowing nutrient circulation [41]. However, whether marine cyanobacteria and heterotrophic bacteria may exchange nutrients through direct nanotube-like membranous structures remains to be established. Nonetheless, the finding that marine cyanobacteria as well as terrestrial bacteria can directly exchange cytoplasmic molecules with each other using nanotubes is important to shed light on the evolution and ecology of bacterial life in the oceanic and terrestrial ecosystems. Furthermore, the discovery that both hereditary and nonhereditary antibiotic resistance may be transferred to neighboring cells by nanotubes [5] implies that the emergence of these direct communication highways may have represented an evolutionary benefit as it would confer a survival advantage.

Mitochondria are the powerhouse and key signaling hubs of eukaryotic cells [42]. The current endosymbiotic theory on the origin of mitochondria implies that millions of years ago a primitive eukaryotic cell “engulfed” an aerobic bacterium, establishing a symbiotic relationship [42]. In 2008, inter-organelle mitochondrial nanotubes were visualized for the first time in cultured African green monkey kidney cells [43]. Mitochondrial nanotunnels are double-membrane protrusions 40–200 nm in diameter and up to 30 μm long, emerging from mitochondria and characterized by limited motility (reviewed in [44]). These intracellular conduits transport mitochondrial matrix and mitochondrial membrane proteins between mitochondria under different stress conditions [44], resembling bacterial nanotubes [44]. As suggested by Picard’s group [44], the presence of an inter-mitochondrial web suggests that nanotubes may represent a “communication system” trait of ancient origins. Future research on the implication of mitochondrial nanotunnels in cell homeostasis may advance our understanding of mitochondrial biology and propagation of mitochondrial signaling under physiological and pathological states. It is worth mentioning that recent evidence shows that mitochondrial genome (mtDNA) nucleoids are transported through the nanotubular mitochondrial network in human, rat and monkey cells through a mitochondrial inner-membrane protein complex MICOS, which links nucleoids to Miro1, a mitochondrial KIF5B receptor, at the endoplasmic reticulum (ER)–mitochondria contact sites [45]. Interestingly, this active transport system transfers mtDNA nucleoids in mitochondria located at the periphery of the cell and is particularly active in the presence of mtDNA mutations [45,46]. The biological significance of the local enrichment of nucleoids in mitochondria located at the peripheral zone of the cells is at present unknown. However, it is tempting to speculate that, under metabolic or genetic stress, mitochondria at the periphery of the cells may be among the first to be transferred and redistributed to other cells through intercellular nanotubes, potentially as an attempt to overcome the stress condition. Further studies are needed to elucidate the biological significance of the intramitochondrial network system and to test the hypothesis that a biological relation may exist between the intramitochondrial network and the intercellular nanotube communications.

## 4. Intercellular Bridges in Fungi: Septal Pores

Fungi can grow as yeast (unicellular), pseudohyphae or as hyphae. Fungi growing as yeast typically lack intercellular bridges, though *Saccharomyces cerevisiae* (but not *Saccharomyces pombe*) can form interspore bridges, composed of the chitosan layer of the spore walls, which provide physical anchoring between adjacent spores within an ascus during sporulation but do not represent an intercellular transport system [47]. Instead, fungi growing as hyphae form intercellular bridges that allow exchange of nuclei and cytoplasmic materials, including mitochondria and vacuoles, between flanking cells through septal pores [13,48]. Though structurally and developmentally different from bacterial and mammalian tunneling nanotubes, the intercellular bridges of filamentous fungi represent a functional analogy with the intercellular nanotubes present in other kingdoms of life, by allowing direct cytoplasmic exchange among cells. In turn, transfer of nutrients through fungal septal pores between cells at the tip of the hyphae and subapical cells would allow the hyphae to expand [49]. Septal pores were first described in 1958 in basidiomycota [50]. Later, it was discovered that the Ascomycota fungi have developed a mechanism to block septal pores using highly refractile particles present on the sides of the septum and known as Woronin bodies [48]. These peroxisome-derived bodies function as a septal plug when hyphae damage or other stresses occur and are aimed at preventing excessive cytoplasmic bleeding [49,51]. In Ascomycetes, the endoplasmic reticulum localizes close to the septal pores, forming a tubular network along the septa, which may play a role in intercellular communication [52]. Furthermore, using *Aspergillus nidulans* as a model system, it has been shown that septal pore permeability is cell cycle regulated to ensure that septal pores are closed during mitosis and reopen during interphase, when the cell cycle regulator kinase NIMA translocates from the nucleus to the septal pore. Notably, NIMA inactivation results in septal pore closure during interphase, demonstrating its critical role in regulating cell cycle-dependent opening of the septal pores [51]. In contrast with Ascomycota, species of the subphylum Agaricomycotina of Basidiomycota lack Woronin bodies and display an endoplasmic reticulum-derived septal pore cap to close the septal pores [53]. Notably, while bacterial nanotubes are transient structures appearing mainly under stress conditions, the intercellular bridges in filamentous fungi are permanent communication systems, similarly to plasmodesmata in plants, which allow cytoplasmic flow in the colony and give turgor to the hyphae [54,55,56], thereby representing an essential structural component of filamentous fungi.

## 5. Plasmodesmata in Algae and Plants

All multicellular organisms have developed specialized systems to communicate, coordinate growth and support the functional specialization of cells and tissues across the organism. Multicellular photosynthetic organisms are the first eukaryotes in which the presence of cytoplasmic bridges between neighbor cells has been demonstrated. In 1879, Eduard Tangle observed cytoplasmic connections between cells in *Strychnos nuxvomica* and described them as protoplasmic bodies “united by thin strands passing through connecting ducts in the walls, which put the cells into connection with each other and so unite them to an entity of higher order” (in [1]). These cytoplasmic bridges were later named “plasmodesmata” by Eduard Strasburger in 1901 [1]. Tangle’s description of plasmodesmata was a prophetic view of the symplasm (or symplast), a word coined by Münch in 1930 to describe the cytoplasmic continuity among cells in higher plants [57]. Decades later, plasmodesmata were also described in green algae *Chara corallina* as simple pores lined by a plasma membrane which link the cytoplasm of neighboring cells [2,14]. Notably, the cells of green algae associate with each other to give rise to multicellular colonial aggregates, which are believed to have been the evolutionary precursors of present-day plants. The simple, ‘open’ plasmodesmata discovered in *Chara corallina* would allow the transfer of nutrients and small-to-medium-sized proteins, while restricting the movement of organelles [2]. In terrestrial plants, plasmodesmata have evolved to connect cells across the cell wall by plasma membrane-delimited bridges approximately 40 nm in diameter containing a central rod of endoplasmic reticulum (ER) called desmotubule, absent in algae, which establishes cytoplasmic continuity between cells [6,15]. The sleeve between the plasma membrane and the ER is occupied by proteins anchored to both sides of the sleeve, thereby creating microchannels within the sleeve [6]. These plasmodesmal microchannels have the ability to shrink and dilate, allowing the selective passage of proteins and RNA as ribonucleoprotein (RNP) complexes that are >20 kDa [16]. While small molecules, such as sucrose, travel between cells via passive mode, it is still not fully clear how larger molecules move through plasmodesmata. From an ultrastructural point of view, callose deposition in the cell wall regulates opening and closing of the plasmodesmal aperture [58]. In addition, plasmodesmal openings are located in pectin-rich regions of the cell wall, indicating that the domains of the cell wall that host plasmodesmata have a specific composition [59]. Recent protein profiling analysis has shown that plasmodesmata accommodate receptor proteins and kinases with unknown ligands and that, after stress, these receptors are specifically recruited in plasmodesmata [60,61], suggesting that plasmodesmata are dynamic structures that may deliver specific signals under different physiological and pathological conditions. In addition, the plasma membrane delimiting the plasmodesmata has a different lipid composition compared to the plasma membrane surrounding the plant cell, being the first highly enriched in sterols and sphingolipids. This lipid composition would contribute to anchoring specific plasmodesmata proteins and may be important for selective protein trafficking [62,63]. For a comprehensive review addressing the details of protein and lipid composition of plasmodesmata, see ref. [64].

Similarly to the septal pores in filamentous fungi, plasmodesmata are not transient communication routes. Plants use these stable structures to coordinate functions and development at a supracellular rather than multicellular level [6,65]. Indeed, plasmodesmata mediate phloem loading and unloading of sugars [66], as well as the passage of hormones, including the growth hormone auxin [67], the stress-induced hormone abscisic acid [68,69,70], and gibberellic acid, a plant hormone regulating growth and development, which has been shown to induce callose hydrolysis and plasmodesmata opening associated with the transport of flowering signals and release of dormancy [71]. The opening and closing of plasmodesmata are dependent on callose synthesis and degradation. The most accepted model is that callose deposition would restrict the cytoplasmic sleeve, thereby closing the plasmodesmata, whereas callose hydrolysis would result in plasmodesmata opening [64]. Notably, plasmodesmal callose deposition and plasmodesmata closure have been observed within 30 min after stress induction mediated by a pathogenic bacterial attack [72]. Callose neosynthesis is induced by pathogen-derived molecules including chitin [73], as well as dsRNAs [74], by activating a common signaling module at plasmodesmata [64]. Importantly, plants that are defective in closing their plasmodesmata in response to pathogens are more susceptible to infection [72,73,75]. Thus, in the context of pathogen attack, the closure of plasmodesmata represents a mechanism of cellular defense which decreases symplastic transport and molecular exchange between adjacent cells, allowing survival of the entire plant. Overall, plasmodesmata are considered essential structures that support the development and differentiation of cell types and tissues in plants; however, notwithstanding their importance, the molecular mechanisms of plasmodesmata biogenesis and dynamics of transport of specific molecules and RNAs remain to be identified.

## 6. Nanotunnels in Invertebrates: Cytonemes in *Drosophila melanogaster*

In the animal kingdom, the first evidence of formation of cell-to-cell tunneling tubes between adjacent cells came from time-lapse observations in developing sea urchin larvae in 1961 [3]. The authors described these tubes as “arms” and suggested that “the force producing arm elongation is connected with growth of the skeleton.” [3]. More than 20 years later (1985), these arms were defined as filopodia supporting the motility and migration of the mesenchyme cells during gastrulation [76]. In 1995, the function of these filopodia was revealed to not be connected to cell locomotion but instead likely to be associated with cell-to-cell signaling during gastrulation [77]. This last hypothesis was substantiated four years later by a study in *Drosophila melanogaster,* which revealed the presence of cellular extensions, named cytonemes, protruding from signaling centers towards receiving cells within the wing imaginal disk [4]. Like the sea urchin embryo filopodia, cytonemes are highly dynamic and contain actin. They extend up to 700 μm with a maximum diameter of 0.2 μm, form within minutes, and display a polarized direction of growth [4]. Notably, ex vivo exposure of explants of *Drosophila* wing imaginal disks to a source of fibroblast growth factor (FGF) triggered the formation of cytonemes. In the same study, membranous protrusions were observed when mouse limb bud cells were exposed to FGF, suggesting that cytonemes are long-range cell–cell communication structures present both in invertebrate and vertebrate organisms [4] (also see Section 7 for cytonemes in vertebrates).

In *Drosophila*, cytonemes protrude from organizing centers expressing Hedgehog (Hh) and Decantaplegic (Dpp)/Bone Morphogenic Protein (BMP), suggesting that these cellular extensions transport morphogens and other signaling molecules, such as Wnt proteins, from signal-producing to signal-receiving and responding cells [4,17]. The molecular mechanisms of cytoneme initiation, growth, morphogen loading and delivery are still not completely clear and further studies are needed to elucidate the dynamics of these membranous protrusions. The prevalent hypothesis is that morphogens might drive actin signaling to promote cytoneme initiation and growth. For example, Hh and its deployment protein Dispatched (Disp), as well as Hh-associated co-receptors, can all trigger cytoneme formation in *Drosophila* (for a comprehensive review on cytoneme initiation, see [17]). Owing to the critical role that morphogens play in embryogenesis, it is plausible that dysfunction of the mechanisms of cytoneme formation, transport and morphogen loading/unloading may result in developmental disorders and tumorigenesis. Intriguingly, in a *Drosophila* cancer model that requires tumor–stroma interactions between epithelial cells and myoblasts in the wing disk, impairment of cytonemes induced by downregulation of cell adhesion proteins (Nrg, Caps), actin-binding proteins (SCAR, dia) or a potassium channel protein (Irk2) reduced the number and length of cytonemes, cell-to-cell signaling and, remarkably, rescued flies from otherwise lethal tumors that model human EGFR overexpression and RET-driven tumors [78].

In the context of human diseases, including cancer, it is worthwhile to point out that the genome of the fly *Drosophila melanogaster* is approximately 60% homologous to the human genome, is less redundant, and 75% of the genes involved in the pathogenesis of human diseases have homologs in flies [79,80]. These genetic characteristics, together with the flies’ short half-life and generation time, and the availability of well-established powerful genetic tools, make *Drosophila melanogaster* a suitable model system to study the molecular mechanisms of human diseases (for more details see [79,80,81,82]). Indeed, key signaling pathways involved in development and in cancer, such as the NOTCH, HEDGEHOG, WNT and RAS pathways, were elucidated in *Drosophila* [81,83,84,85]. Thus, given the high degree of evolutionary conservation between *Drosophila melanogaster* and *Homo sapiens* proteins, future studies aimed at understanding cytoneme biogenesis and biology in *Drosophila* may lead to a better comprehension of the biology of tunneling nanotubes in mammals under physiological and pathological conditions.

## 7. Nanotubes in Vertebrates

In the last two decades, several studies have documented cell-to-cell interactions mediated by plasma membrane nanotubular structures in vertebrates. As discussed below, some of these membranous nanotubes mediate transport of signaling molecules (cytonemes and airinemes), while others can transport both signaling molecules and organelles (TNTs and dendritic nanotubes, DNTs). These cellular protrusions can be further subclassified into closed-ended structures (cytonemes, airinemes, and DNTs) and open-ended membranous extensions (TNTs).

### 7.1. Cytonemes

As discussed in Section 6, cytonemes were first described in *Drosophila melanogaster* and in mouse limb bud cells in 1999 [4]. Though most of the research on cytoneme function has been performed in *Drosophila*, recent studies have highlighted the role of cytoneme-mediated transport of morphogens, including members of the Wnt family, during vertebrate development. The Wnt family of signaling proteins affects organ development by regulating which cells undergo differentiation, when cells migrate, and cell cycle progression [86]. In developing tissues, few cells synthetize Wnt proteins, while most of the cells respond to Wnt signaling proteins [86]. In zebrafish embryos, cytoneme-mediated transport of Wnt5b–Ror2 affects gastrulation [87]. Wnt8a, another member of the Wnt family, binds and activates Ror-2, resulting in activation of the planar cell polarity (PCP) pathway and Cdc42, which promotes the outgrowth of signaling cytonemes [88]. Wnt8a is transported through these cytonemes to the effector/responding cells where it binds to the β-catenin co-receptor Lrp6 in zebrafish fibroblasts and HEK293T human embryonic kidney cells, activating the β-catenin pathway. In turn, activated β-catenin signaling regulates neural plate patterning in zebrafish and proliferation in human gastric cancer cells [88,89], indicating that cytonemes are a relevant transport mechanism for the Wnt family proteins in vertebrates.

In addition to Wnt proteins, a recent study demonstrated the importance of cell-to-cell communication mediated by cytonemes for the delivery of the sonic hedgehog (SHH) morphogen in developing mouse neural tubes [90]. Disruption of cytoneme function by mutation of actin motor myosin 10 (MYO10) reduced both SHH and Wnt neuronal signaling [90]. Notably, depletion of SHH modified the cell fate of neuronal cells and impaired neurodevelopment in mammals [90]. Cytonemes are also implicated in transporting Notch between keratinocytes in zebrafish, and inhibition of keratinocytes’ cytonemes reduces the levels of Notch protein in undifferentiated keratinocytes, causing defective differentiation and increased cell proliferation [91], suggesting that deregulation of cytoneme-mediated signaling in keratinocytes may be one of the mechanisms underlining skin diseases in humans.

Taken together, these studies demonstrate the importance of cytoneme-mediated transport of morphogens during vertebrate development.

### 7.2. Airinemes

Specialized membrane projections with vesicles at the tip were described in zebrafish in 2015 and termed “airinemes” [18]. The formation of these projections has been implicated in the characteristic “zebra” stripe patterning of zebrafish. The light/yellow stripes of the zebrafish contain two cell types, known as xanthophores and iridophores. While xanthophores repel melanophores, i.e., the cells present in the dark stripes, xanthoblasts produce airinemes that enter in contact with melanophores and melanoblasts and transduce Delta–Notch signaling which causes melanophores to migrate from yellow stripes to neighborhood dark stripes [18]. Similarly to cytonemes, airinemes contain F-actin. However, while cytonemes are mainly straight projections, airinemes have intricate trajectories and transport vesicles of approximately 1.7 μm in diameter, which is larger than the 30–200 nm particles transported by cytonemes. In addition, airinemes may also contain tubulin, unlike cytonemes [18]. Macrophages were later demonstrated to be involved in aireneme-mediated formation of zebrafish skin stripes [92]. Indeed, macrophages promoted airineme extension from xanthoblasts as well as vesicle deposition on melanophores. Depletion of macrophages prevented melanophores from migrating and gave rise to dark stripes, consistently with the pivotal role of macrophages in relaying long-distance signals between signal producers (xanthoblasts) and signal receivers (melanophores) [92]. Further studies identified these airineme-pulling macrophages as a specific subpopulation known as metaphocytes [93], which mediate airineme signaling and, in contrast with other macrophages, express high levels of matrix metalloproteinase-9 (MMP-9) [19]. By digesting the extracellular matrix, MMP-9 would allow metaphocytes to migrate deeper and faster into the hypodermis where xanthoblasts are localized [19]. Finally, it has been recently reported that the interaction between the extracellular domain of CD44 on macrophages’ plasma membrane and airineme vesicles is crucial for airineme-mediated signaling [20]. Metaphocytes lacking a CD44 extracellular domain showed defective cell adhesion properties, resulting in reduced airineme length and defects in the development of the zebrafish pigment pattern [20]. To date, the existence of airinemes has been described only in zebrafish. Given the pivotal role of airinemes in transporting signaling molecules involved in development and cell migration, it will be interesting to investigate whether these signaling membranous protrusions also exist in higher vertebrates.

### 7.3. Tunneling Nanotubes (TNTs)

In 2004, Rustom et al. [94] described open-ended cellular protrusions in rat pheochromocytoma PC12 cells, human embryonic kidney cells and rat kidney cells. These protrusions, termed tunneling nanotubes (TNTs), had a diameter of up to 200 nm and a length covering several cell diameters. It was later demonstrated that TNTs can reach a diameter of >700 nm (thick TNTs [29]; for a comprehensive overview of TNT structure see refs. [27,28]). Similarly to cytonemes, TNTs are mainly straight projections and contain F-actin. In addition, their membrane is continuous with the membrane of the connected cell/s [94], similarly to plasmodesmata in the plant kingdom [6,21]. Initial observations demonstrated that organelles and components of the plasma membrane in the form of membrane vesicles could be transferred between cells through TNTs [21]. It was later discovered that TNTs could transfer mitochondria between neonatal rat cardiomyocytes and endothelial progenitor cells [95], as well as endoplasmic reticulum and Golgi apparatus [22]. Though TNTs have been observed in cells under no-stress conditions [96], the formation of these intercellular channels seems to occur more often under stress conditions, including hypoxia, ultraviolet and ionizing radiation [97,98], and during viral infection. Notably, TNTs have been recently described in zebrafish embryos [99], thus demonstrating that TNTs are not mammal-specific intercellular communication systems, but likely vertebrate-conserved.

**Figure 1:**
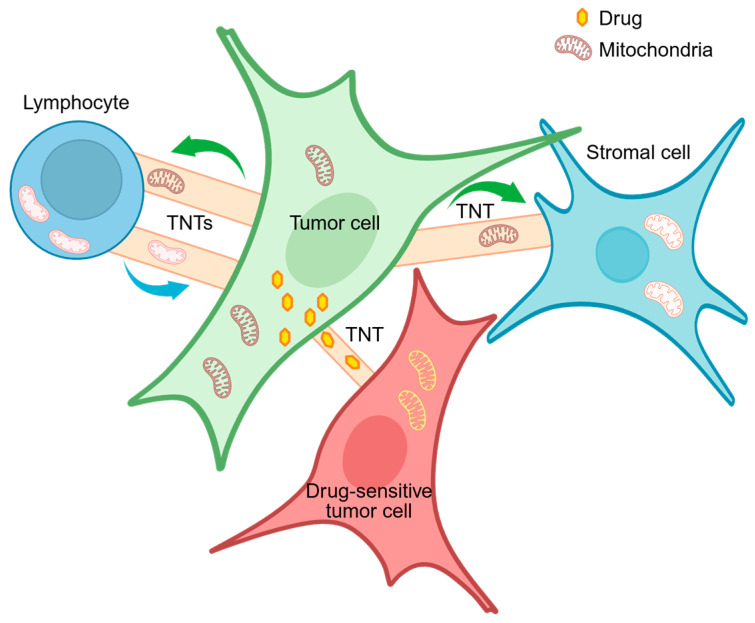
Schematic of the TNT-mediated cell-to-cell transfer of mitochondria and drugs between tumor and non-tumor cells. In the tumor microenvironment, mitochondria can be exchanged between tumor cells and T lymphocytes via TNTs in a bidirectional way, promoting T cell exhaustion. TNTs can also transfer drugs from chemoresistant tumor cells to chemosensitive tumor cells. Created in https://BioRender.com.

The horizontal or intercellular transfer of mitochondria through TNTs has received much attention in recent years [100], owing to the importance of these organelles as the energetic powerhouse of the cell and as signaling hubs [101]. Mitochondria are shuttled through TNT channels using Rho-GTPase Miro1 to enter the cytoplasm of the receiving cell [102]. The horizontal transfer of mitochondria occurs between cells of the same type as well as between different cell types. In this context, a recent study has shown that glioblastoma cells use TNTs as a communication route with the T cells present in the tumor microenvironment to hijack healthy mitochondria from T cells for their own advantage [103] (Figure 1). Glioblastoma is a highly aggressive cancer type characterized by an immune evasion mechanism related to enrichment of immunosuppressive myeloid cells and exhausted T cells in the tumor microenvironment [104]. The transfer of healthy mitochondria from T cells to glioblastoma cells promoted a decrease in mitochondrial reactive oxygen species (ROS) in tumor cells and was associated with reduced viability of T cells, thus supporting immune evasion [103]. In human bladder cancer cells, mitochondrial transfer via TNTs between cancer cells at different stages and grades was associated with gain of invasive properties by cells with low invasive potential after transfer of mitochondria from cells with high invasive ability [105], consistent with previous studies showing that mitochondria may modulate cancer cell migration and invasion [101,106,107]. Furthermore, in a mouse model of melanoma, Miro1-mediated TNTs allowed transfer of mitochondrial DNA (mtDNA) from mtDNA-full stromal cells to mtDNA-deficient melanoma cells, thereby restoring mitochondrial respiration and oxidative phosphorylation in cancer cells and promoting melanoma growth [108]. Finally, a recent study provides evidence of a TNT-dependent mechanism of cancer immune evasion through transfer of mitochondria with mutant mtDNA from cancer cells to tumor-infiltrating lymphocytes (TILs) [109]. While mitochondria in TILs normally undergo mitophagy, a quality control mechanism to remove damaged mitochondria from the cells, mitochondria with mutant mtDNA acquired from cancer cells did not undergo mitophagy, resulting in senescence and metabolic alterations, which decreased T cell effector functions and promoted T cell exhaustion *in vivo* and *in vitro*, thereby impairing antitumor immunity [109]. These results identify a new mechanism of immune evasion mediated by cancer-to-immune cell intercellular transfer of mutant mtDNA through TNTs, and provide a rationale for the observed poor prognostic factor of mtDNA mutations for therapy with immune checkpoint inhibitors (PD-1 blockade) in patients with lung cancer or melanoma [109].

In addition to being implicated in immune evasion, TNTs have also been suggested to play a role in promoting and propagating drug resistance. Following exposure to chemotherapeutic drugs, Jurkat T-cell leukemic cells transferred ROS-rich mitochondria to mesenchymal stem cells through TNTs, thereby boosting their own survival by redistributing ROS [110]. In pancreatic adenocarcinoma cell lines and ovarian cancer cells, exposure to increasing doses of doxorubicin caused a dose-dependent increase in formation of TNTs, which redistributed the drug from drug-resistant to drug-sensitive cells [23,24]. Interestingly, transport of the drug resulted in the induction of apoptosis in the receiving sensitive cells [23,24], suggesting that efflux and redistribution of chemotherapeutic drugs through TNTs may favor chemoresistant, often more invasive, cancer cells, thereby supporting the establishment of a chemoresistant cancer niche.

In 2008, Sowinski et al. [111] demonstrated that T cells communicate with each other through TNTs and that this physical type of connection allows rapid viral spread of the retrovirus HIV-1, the causative agent of acquired immune deficiency syndrome (AIDS). It was later shown that human macrophages infected with HIV displayed an increased formation of TNTs containing HIV particles, indicating that TNT-mediated communication may be used by HIV to spread to neighboring cells [112].

In addition to HIV-1, other viruses have been shown to use TNTs for dissemination (for an exhaustive review see [113]). Human T-cell leukemia virus type 1 (HTLV-1) uses T cells’ TNTs for transmission and dissemination [114]. In particular, the HTLV’s protein p8 can increase the intercellular conduits between CD4+ memory T cells, promoting viral diffusion while escaping the host’s immune surveillance [114]. Influenza virus type A also uses TNTs to spread from infected to uninfected cells [115]. These TNTs contain several viral proteins, viral nucleoproteins and the influenza virus genome [115]. Two studies published in 2022 have demonstrated that severe acute respiratory syndrome coronavirus 2 (SARS-CoV-2), the pathogen responsible for the COVID-19 pandemic, enhances infection between permissive cells and spreads between permissive infected cells and non-permissive neuronal cells through TNTs [116,117]. Spreading through TNTs would also shield SARS-CoV-2 from immune recognition, thus representing an effective immune evasion strategy [117]. Djurkovic et al. [118] have recently shown that infection of human macrophages with Ebola virus, a negative-strand RNA virus responsible for the frequently fatal Ebola disease, promotes the formation of TNTs of different lengths and diameter and that only TNTs containing tubulin and transferring organelles also transport viral proteins and genomes [118]. Interestingly, TNTs promote the transfer of nucleocapsids between the host’s cells in the absence of a live virus, providing evidence for a pathway of virus dissemination alternative to the emergence of nascent virions from the cell surface [118]. Finally, the Zika virus (ZIKV), a mosquito-borne positive-strand RNA virus, which causes congenital Zika syndrome, induces the formation of TNTs in placental trophoblasts, thereby promoting the transfer of viral proteins, RNA and cell mitochondria to non-infected cells [119]. The viral protein NS1 has been implicated in the induction of TNTs. Intriguingly, ZIKV infection or NS1 expression induced the movement of mitochondria via TNTs from healthy trophoblast cells to ZIKV-infected cells. This mitochondria hijacking would support ZIKV propagation and survival [119].

Recent studies have shown that TNTs play an important role in the nervous system, both in physiological and pathological conditions. From a physiological point of view, TNT formation has been implicated in neurogenesis. TNT formation has been observed between immature hippocampal neurons and astrocytes [120]. These tunneling protrusions mediated the propagation of electric and calcium signals transiently for a few hours and ceased to transfer signals after 24 h, likely due to neuronal differentiation [120]. In a pathological context, such as neurodegeneration, the formation of TNTs has been implicated in the transport of neurotoxic protein aggregates. Neurodegenerative diseases such as Alzheimer’s, Huntington’s and Parkinson’s diseases are characterized by the accumulation of protein aggregates, which eventually cause neuronal death [121]. Infectious prion PrPSc (causing Creutzfeldt–Jakob disease [122]), huntingtin protein (causing Huntington’s disease [123]), α-synuclein (presynaptic neuronal protein linked to Parkinson’s disease [124]), and Tau and β-amyloid proteins (linked to Alzheimer’s disease [125,126]) are among the protein aggregates shown to propagate from cell to cell via TNTs in the central nervous system (a topic extensively reviewed in [26,127,128,129]). Using a mouse neuronal cell line of catecholaminergic origin, Gousset et al. [130] demonstrated that prion PrPSc spreads via TNTs not only between neurons but also from bone marrow-derived dendritic cells to primary neurons, indicating that TNT-mediated cell-to-cell communication may allow neurons to transfer prions from the periphery to the central nervous system. TNT-mediated transport of neurotoxic protein aggregates, like prion PrPSc and Tau fibrils, was also observed between astrocytes and neurons [128,131,132]. In 2023, Scheiblich et al. [25] and Chakraborty et al. [133] demonstrated that neurons use TNTs to transfer α-synuclein and Tau aggregates to the microglia (immune cells of the brain) which, in turn, transfer functional mitochondria to neurons, ameliorating neuronal health, thus suggesting that the transport of functional mitochondria between neurons and microglia may help slowing the progression of neurodegenerative diseases [25]. More recently, Chakraborty et al. [134] reported that impaired autophagy in neurons represents a key driver inducing the transfer of α-synuclein aggregates to microglia, which, in turn, activate the autophagic route to degrade the protein aggregates.

Overall, TNTs have emerged as key conduits of signaling molecules and pathogens, a route for efflux and redistribution of chemotherapeutic drugs and for the transfer of functional and dysfunctional organelles, including mitochondria, in a cell context-dependent manner. A deeper understanding of TNT-mediated communications may lead to a better comprehension of the underlying mechanisms of pathogen transmission, as well as neurodegeneration and cancer development and progression, with potential future implications in the therapies of these diseases.

### 7.4. Dendritic Nanotubes (DNTs)

In 2025, discovery of a new type of neural network within the brain, made of dendritic nanotubes, opened a new chapter into the biology, physiology and anatomy of brain connectivity [30]. Specialized junctions known as synapses have long been thought to be the exclusive communication system between neurons. Chang et al. [30] discovered that a filopodium from a neuron’s dendrite enters in contact with another dendrite belonging to a neighboring neuron, forming membrane-to-membrane vesicular contacts both in mouse and human brain tissue. These protrusions were termed by the authors dendritic nanotubes (DNTs), which, unlike TNTs, are not open-ended tunneling systems of communication and do not connect cell bodies. DNTs are characterized by an average length of 3 μm, a diameter less than 400 nm and a lifetime of a few hours. Like TNTs, they contain actin. DNTs have been shown to transport calcium ions, small molecules and human amyloid-β (Aβ). The formation of Aβ plaques is a hallmark of Alzheimer’s disease. In a mouse model of Alzheimer’s disease, DNT density increased before the formation of Aβ plaques in the medial prefrontal cortex. With time, accumulated Aβ reduced DNTs [30]. The authors suggested a pathological negative feedback loop, where the loss of DNPs leads to the accumulation of Aβ plaques, thereby further reducing DNT formation [30]. According to mathematical models, DNT-mediated transport of Aβ recapitulated early amyloidosis [30], thus unraveling a new mechanism in the pathogenesis of Alzheimer’s disease. In addition, the DNT system of neuronal connectivity may operate in parallel with synapses to transmit electrical signals in the form of calcium ions. Further studies are needed to understand the biology and structure of these intriguing nanotubular systems of intercellular communication in the brain and their implication in the pathogenesis of neurodegenerative diseases.

## 8. Conclusions

The discovery of the existence of a network of cell-to-cell membranous nanotubes allowing long-distance transfer of cytoplasmic molecules and organelles has brought our knowledge of the mechanisms of signal propagation to a higher level of complexity. The presence of similar intercellular membrane protrusions in prokaryotic and eukaryotic cells supports the hypothesis of an ancestral origin of these communication structures conserved during evolution. Alternatively, these cell-to-cell nanotubes may have evolved independently in prokaryotic and eukaryotic organisms as an example of convergent evolution to adapt to similar constraints, such as to thrive under stress conditions.

Evidence on the functional significance of intercellular nanotubes in different kingdoms of life is rapidly increasing and supports a pivotal role of these membrane protrusions in conveying morphogenic, stress-response and survival signals. The finding that mitochondria, the eukaryotic organelles of prokaryotic origin defined by Herrmann as “the beating heart of the eukaryotic cell” [135], can form an intracellular network of mitochondria-to-mitochondria nanotubes exchanging nutrients and signals [44], just like bacteria-to-bacteria and eukaryotic-to-eukaryotic cells, is intriguing and suggests their potential relevance in cell homeostasis and mitochondrial pathophysiology. Finally, as nanotube-mediated intercellular signaling is implicated in cancer development and drug resistance, as well as in the pathogenesis of neurodegenerative diseases and the spreading of viral infections, the potential exists that targeting these structures might be used in therapy in the future.

## Figures and Tables

**Table 1:** Nanotube’s structure and cargo transport in 5 kingdoms of life.

Kingdom	Structure	Diameter	Length	Lifetime	Cargo
Bacteria	Nanotubes	30–160 nm	100–600 nm	Transient	Metabolites, proteins, small RNAs [5,10,11,12]
Fungi	Septal pores	50–500 nm	n.a.	Permanent	Proteins, RNAs, organelles, nuclei, vesicles [13]
Algae	Plasmodesmata	Up to 20 nm	40 μm–1 mm	Permanent	Nutrients, proteins [2,14]
Plants	Plasmodesmata	2–40 nm	Whole plant (symplast)	Permanent	Ions, sugars, hormones, proteins, RNAs, pathogens [6,15,16]
Animals	Cytonemes	200 nm	≤100 μm	Transient	Morphogens, signaling vesicles, ligand–receptor complexes at contact sites [4,17]
	Airinemes	≤2 mm	250 μm	Transient	Signaling vesicles, proteins [18,19,20]
	Tunneling nanotubes (TNTs)	50–200 nm (up to 1 μm for thick TNTs)	≤200 μm	Transient	Mitochondria, endoplasmic reticulum, Golgi apparatus, proteins, protein aggregates, drugs, pathogens, signaling vesicles [21,22,23,24,25,26,27,28,29]
	Dendritic nanotubes (DNTs)	≤400 nm	3 μm	Transient	Ions, small molecules, proteins [30]

## Data Availability

No new data were created or analyzed in this study. Data sharing is not applicable to this article.

## References

[1] Oparka K.J., Roberts A.G. (2001). Plasmodesmata. A not so open-and-shut case. Plant Physiol..

[2] Franceschi V.R., Ding B., Lucas W.J. (1994). Mechanism of plasmodesmata formation in characean algae in relation to evolution of intercellular communication in higher plants. Planta.

[3] Gustafson T., Wolpert L. (1961). Cellular mechanisms in the morphogenesis of the sea urchin larva. The formation of arms. Exp. Cell Res..

[4] Ramirez-Weber F.A., Kornberg T.B. (1999). Cytonemes: Cellular processes that project to the principal signaling center in Drosophila imaginal discs. Cell.

[5] Dubey G.P., Ben-Yehuda S. (2011). Intercellular nanotubes mediate bacterial communication. Cell.

[6] Lucas W.J., Lee J.Y. (2004). Plasmodesmata as a supracellular control network in plants. Nat. Rev. Mol. Cell Biol..

[7] Bleichrodt R.J., van Veluw G.J., Recter B., Maruyama J., Kitamoto K., Wosten H.A. (2012). Hyphal heterogeneity in Aspergillus oryzae is the result of dynamic closure of septa by Woronin bodies. Mol. Microbiol..

[8] Lenardon M.D., Munro C.A., Gow N.A. (2010). Chitin synthesis and fungal pathogenesis. Curr. Opin. Microbiol..

[9] Faulkner C. (2018). Plasmodesmata and the symplast. Curr. Biol..

[10] Pande S., Shitut S., Freund L., Westermann M., Bertels F., Colesie C., Bischofs I.B., Kost C. (2015). Metabolic cross-feeding via intercellular nanotubes among bacteria. Nat. Commun..

[11] Stempler O., Baidya A.K., Bhattacharya S., Malli Mohan G.B., Tzipilevich E., Sinai L., Mamou G., Ben-Yehuda S. (2017). Interspecies nutrient extraction and toxin delivery between bacteria. Nat. Commun..

[12] Patel N., Yamada Y., Azam F. (2021). Bacterial Nanotubes as Intercellular Linkages in Marine Assemblages. Front. Mar. Sci..

[13] Sudbery P., Gow N., Berman J. (2004). The distinct morphogenic states of Candida albicans. Trends Microbiol..

[14] Cook M., Graham L., Botha C., Lavin C. (1997). Comparative ultrastructure of plasmodesmata of Chara and selected bryophytes: Toward an elucidation of the evolutionary origin of plant plasmodesmata. Am. J. Bot..

[15] Tilney L.G., Cooke T.J., Connelly P.S., Tilney M.S. (1991). The structure of plasmodesmata as revealed by plasmolysis, detergent extraction, and protease digestion. J. Cell Biol..

[16] Noueiry A.O., Lucas W.J., Gilbertson R.L. (1994). Two proteins of a plant DNA virus coordinate nuclear and plasmodesmal transport. Cell.

[17] Daly C.A., Hall E.T., Ogden S.K. (2022). Regulatory mechanisms of cytoneme-based morphogen transport. Cell. Mol. Life Sci..

[18] Eom D.S., Bain E.J., Patterson L.B., Grout M.E., Parichy D.M. (2015). Long-distance communication by specialized cellular projections during pigment pattern development and evolution. eLife.

[19] Bowman R.L., Wang D., Eom D.S. (2023). A macrophage subpopulation promotes airineme-mediated intercellular communication in a matrix metalloproteinase-9 dependent manner. Cell Rep..

[20] Bowman R.L., Kim J., Parsons M.J., Eom D.S. (2025). CD44 facilitates adhesive interactions in airineme-mediated intercellular signaling. Front. Cell Dev. Biol..

[21] Nakajima K., Sena G., Nawy T., Benfey P.N. (2001). Intercellular movement of the putative transcription factor SHR in root patterning. Nature.

[22] Wang Y., Cui J., Sun X., Zhang Y. (2011). Tunneling-nanotube development in astrocytes depends on p53 activation. Cell Death Differ..

[23] Sarkari A., Lou E. (2024). Do tunneling nanotubes drive chemoresistance in solid tumors and other malignancies?. Biochem. Soc. Trans..

[24] Desir S., O’Hare P., Vogel R.I., Sperduto W., Sarkari A., Dickson E.L., Wong P., Nelson A.C., Fong Y., Steer C.J. (2018). Chemotherapy-Induced Tunneling Nanotubes Mediate Intercellular Drug Efflux in Pancreatic Cancer. Sci. Rep..

[25] Scheiblich H., Eikens F., Wischhof L., Opitz S., Jungling K., Cserep C., Schmidt S.V., Lambertz J., Bellande T., Posfai B. (2024). Microglia rescue neurons from aggregate-induced neuronal dysfunction and death through tunneling nanotubes. Neuron.

[26] Palese F., Rakotobe M., Zurzolo C. (2025). Transforming the concept of connectivity: Unveiling tunneling nanotube biology and their roles in brain development and neurodegeneration. Physiol. Rev..

[27] Medina L.Y., Serda R.E. (2024). Intercellular Communication Through Microtubular Highways. Results and Problems in Cell Differentiation.

[28] Cordero Cervantes D., Zurzolo C. (2021). Peering into tunneling nanotubes-The path forward. EMBO J..

[29] Onfelt B., Nedvetzki S., Benninger R.K., Purbhoo M.A., Sowinski S., Hume A.N., Seabra M.C., Neil M.A., French P.M., Davis D.M. (2006). Structurally distinct membrane nanotubes between human macrophages support long-distance vesicular traffic or surfing of bacteria. J. Immunol..

[30] Chang M., Krussel S., Parajuli L.K., Kim J., Lee D., Merodio A., Kwon J., Okabe S., Kwon H.B. (2025). Intercellular communication in the brain through a dendritic nanotubular network. Science.

[31] Alegado R.A., King N. (2014). Bacterial influences on animal origins. Cold Spring Harb. Perspect. Biol..

[32] Mojzsis S.J., Arrhenius G., McKeegan K.D., Harrison T.M., Nutman A.P., Friend C.R. (1996). Evidence for life on Earth before 3,800 million years ago. Nature.

[33] Gaudin M., Gauliard E., Schouten S., Houel-Renault L., Lenormand P., Marguet E., Forterre P. (2013). Hyperthermophilic archaea produce membrane vesicles that can transfer DNA. Environ. Microbiol. Rep..

[34] Wei X., Vassallo C.N., Pathak D.T., Wall D. (2014). Myxobacteria produce outer membrane-enclosed tubes in unstructured environments. J. Bacteriol..

[35] Dubey G.P., Malli Mohan G.B., Dubrovsky A., Amen T., Tsipshtein S., Rouvinski A., Rosenberg A., Kaganovich D., Sherman E., Medalia O. (2016). Architecture and Characteristics of Bacterial Nanotubes. Dev. Cell.

[36] Bhattacharya S., Baidya A.K., Pal R.R., Mamou G., Gatt Y.E., Margalit H., Rosenshine I., Ben-Yehuda S. (2019). A Ubiquitous Platform for Bacterial Nanotube Biogenesis. Cell Rep..

[37] Pal R.R., Baidya A.K., Mamou G., Bhattacharya S., Socol Y., Kobi S., Katsowich N., Ben-Yehuda S., Rosenshine I. (2019). Pathogenic *E. coli* Extracts Nutrients from Infected Host Cells Utilizing Injectisome Components. Cell.

[38] Pospisil J., Vitovska D., Kofronova O., Muchova K., Sanderova H., Hubalek M., Sikova M., Modrak M., Benada O., Barak I. (2020). Bacterial nanotubes as a manifestation of cell death. Nat. Commun..

[39] Flombaum P., Gallegos J.L., Gordillo R.A., Rincon J., Zabala L.L., Jiao N., Karl D.M., Li W.K., Lomas M.W., Veneziano D. (2013). Present and future global distributions of the marine Cyanobacteria Prochlorococcus and Synechococcus. Proc. Natl. Acad. Sci. USA.

[40] Angulo-Canovas E., Bartual A., Lopez-Igual R., Luque I., Radzinski N.P., Shilova I., Anjur-Dietrich M., Garcia-Jurado G., Ubeda B., Gonzalez-Reyes J.A. (2024). Direct interaction between marine cyanobacteria mediated by nanotubes. Sci. Adv..

[41] Christie-Oleza J.A., Sousoni D., Lloyd M., Armengaud J., Scanlan D.J. (2017). Nutrient recycling facilitates long-term stability of marine microbial phototroph-heterotroph interactions. Nat. Microbiol..

[42] Atlante A., Valenti D. (2023). Mitochondria Have Made a Long Evolutionary Path from Ancient Bacteria Immigrants within Eukaryotic Cells to Essential Cellular Hosts and Key Players in Human Health and Disease. Curr. Issues Mol. Biol..

[43] Bowes T., Gupta R.S. (2008). Novel mitochondrial extensions provide evidence for a link between microtubule-directed movement and mitochondrial fission. Biochem. Biophys. Res. Commun..

[44] Vincent A.E., Turnbull D.M., Eisner V., Hajnoczky G., Picard M. (2017). Mitochondrial Nanotunnels. Trends Cell Biol..

[45] Qin J., Guo Y., Xue B., Shi P., Chen Y., Su Q.P., Hao H., Zhao S., Wu C., Yu L. (2020). ER-mitochondria contacts promote mtDNA nucleoids active transportation via mitochondrial dynamic tubulation. Nat. Commun..

[46] Eisner V., Picard M., Hajnoczky G. (2018). Mitochondrial dynamics in adaptive and maladaptive cellular stress responses. Nat. Cell Biol..

[47] Coluccio A., Neiman A.M. (2004). Interspore bridges: A new feature of the Saccharomyces cerevisiae spore wall. Microbiology.

[48] Bloemendal S., Kuck U. (2013). Cell-to-cell communication in plants, animals, and fungi: A comparative review. Naturwissenschaften.

[49] Trinci A.P., Collinge A.J. (1973). Structure and plugging of septa of wild type and spreading colonial mutants of Neurospora crassa. Arch. Mikrobiol..

[50] Girbardt M. (1958). About the substructure of *Polystictus versicolor* L.. Arch. Mikrobiol..

[51] Shen K.F., Osmani A.H., Govindaraghavan M., Osmani S.A. (2014). Mitotic regulation of fungal cell-to-cell connectivity through septal pores involves the NIMA kinase. Mol. Biol. Cell.

[52] Maruyama J., Kikuchi S., Kitamoto K. (2006). Differential distribution of the endoplasmic reticulum network as visualized by the BipA-EGFP fusion protein in hyphal compartments across the septum of the filamentous fungus, Aspergillus oryzae. Fungal Genet. Biol..

[53] van Peer A.F., Wang F., van Driel K.G., de Jong J.F., van Donselaar E.G., Muller W.H., Boekhout T., Lugones L.G., Wosten H.A. (2010). The septal pore cap is an organelle that functions in vegetative growth and mushroom formation of the wood-rot fungus Schizophyllum commune. Environ. Microbiol..

[54] Abadeh A., Lew R.R. (2013). Mass flow and velocity profiles in Neurospora hyphae: Partial plug flow dominates intra-hyphal transport. Microbiology.

[55] Roper M., Lee C., Hickey P.C., Gladfelter A.S. (2015). Life as a moving fluid: Fate of cytoplasmic macromolecules in dynamic fungal syncytia. Curr. Opin. Microbiol..

[56] Jedd G., Pieuchot L. (2012). Multiple modes for gatekeeping at fungal cell-to-cell channels. Mol. Microbiol..

[57] Munch E. (1930). Die Stoffbewegungen in der Pflanze.

[58] Gaudioso-Pedraza R., Beck M., Frances L., Kirk P., Ripodas C., Niebel A., Oldroyd G.E.D., Benitez-Alfonso Y., de Carvalho-Niebel F. (2018). Callose-Regulated Symplastic Communication Coordinates Symbiotic Root Nodule Development. Curr. Biol..

[59] Paterlini A., Sechet J., Immel F., Grison M.S., Pilard S., Pelloux J., Mouille G., Bayer E.M., Voxeur A. (2022). Enzymatic fingerprinting reveals specific xyloglucan and pectin signatures in the cell wall purified with primary plasmodesmata. Front. Plant Sci..

[60] Fernandez-Calvino L., Faulkner C., Walshaw J., Saalbach G., Bayer E., Benitez-Alfonso Y., Maule A. (2011). Arabidopsis plasmodesmal proteome. PLoS ONE.

[61] Grison M.S., Kirk P., Brault M.L., Wu X.N., Schulze W.X., Benitez-Alfonso Y., Immel F., Bayer E.M. (2019). Plasma Membrane-Associated Receptor-like Kinases Relocalize to Plasmodesmata in Response to Osmotic Stress. Plant Physiol..

[62] Yan D., Yadav S.R., Paterlini A., Nicolas W.J., Petit J.D., Brocard L., Belevich I., Grison M.S., Vaten A., Karami L. (2019). Sphingolipid biosynthesis modulates plasmodesmal ultrastructure and phloem unloading. Nat. Plants.

[63] Liu N.J., Zhang T., Liu Z.H., Chen X., Guo H.S., Ju B.H., Zhang Y.Y., Li G.Z., Zhou Q.H., Qin Y.M. (2020). Phytosphinganine Affects Plasmodesmata Permeability via Facilitating PDLP5-Stimulated Callose Accumulation in Arabidopsis. Mol. Plant.

[64] Tee E.E., Faulkner C. (2024). Plasmodesmata and intercellular molecular traffic control. New Phytol..

[65] Lucas W.J., Wolf S. (1993). Plasmodesmata: The intercellular organelles of green plants. Trends Cell Biol..

[66] Liesche J., Gao C., Binczycki P., Andersen S.R., Rademaker H., Schulz A., Martens H.J. (2019). Direct Comparison of Leaf Plasmodesma Structure and Function in Relation to Phloem-Loading Type. Plant Physiol..

[67] Gao C., Liu X., De Storme N., Jensen K.H., Xu Q., Yang J., Liu X., Chen S., Martens H.J., Schulz A. (2020). Directionality of Plasmodesmata-Mediated Transport in Arabidopsis Leaves Supports Auxin Channeling. Curr. Biol..

[68] Kitagawa M., Tomoi T., Fukushima T., Sakata Y., Sato M., Toyooka K., Fujita T., Sakakibara H. (2019). Abscisic Acid Acts as a Regulator of Molecular Trafficking through Plasmodesmata in the Moss Physcomitrella patens. Plant Cell Physiol..

[69] Mehra P., Pandey B.K., Melebari D., Banda J., Leftley N., Couvreur V., Rowe J., Anfang M., De Gernier H., Morris E. (2022). Hydraulic flux-responsive hormone redistribution determines root branching. Science.

[70] Tylewicz S., Petterle A., Marttila S., Miskolczi P., Azeez A., Singh R.K., Immanen J., Mahler N., Hvidsten T.R., Eklund D.M. (2018). Photoperiodic control of seasonal growth is mediated by ABA acting on cell-cell communication. Science.

[71] Rinne P.L., Welling A., Vahala J., Ripel L., Ruonala R., Kangasjarvi J., van der Schoot C. (2011). Chilling of dormant buds hyperinduces FLOWERING LOCUS T and recruits GA-inducible 1,3-beta-glucanases to reopen signal conduits and release dormancy in Populus. Plant Cell.

[72] Xu B., Cheval C., Laohavisit A., Hocking B., Chiasson D., Olsson T.S.G., Shirasu K., Faulkner C., Gilliham M. (2017). A calmodulin-like protein regulates plasmodesmal closure during bacterial immune responses. New Phytol..

[73] Faulkner C., Petutschnig E., Benitez-Alfonso Y., Beck M., Robatzek S., Lipka V., Maule A.J. (2013). LYM2-dependent chitin perception limits molecular flux via plasmodesmata. Proc. Natl. Acad. Sci. USA.

[74] Huang C., Sede A.R., Elvira-Gonzalez L., Yan Y., Rodriguez M.E., Mutterer J., Boutant E., Shan L., Heinlein M. (2023). dsRNA-induced immunity targets plasmodesmata and is suppressed by viral movement proteins. Plant Cell.

[75] Wang X., Sager R., Cui W., Zhang C., Lu H., Lee J.Y. (2013). Salicylic acid regulates Plasmodesmata closure during innate immune responses in Arabidopsis. Plant Cell.

[76] Karp G.C., Solursh M. (1985). Dynamic activity of the filopodia of sea urchin embryonic cells and their role in directed migration of the primary mesenchyme in vitro. Dev. Biol..

[77] Miller J., Fraser S.E., McClay D. (1995). Dynamics of thin filopodia during sea urchin gastrulation. Development.

[78] Fereres S., Hatori R., Hatori M., Kornberg T.B. (2019). Cytoneme-mediated signaling essential for tumorigenesis. PLoS Genet..

[79] Ugur B., Chen K., Bellen H.J. (2016). Drosophila tools and assays for the study of human diseases. Dis. Model. Mech..

[80] Mirzoyan Z., Sollazzo M., Allocca M., Valenza A.M., Grifoni D., Bellosta P. (2019). *Drosophila melanogaster*: A Model Organism to Study Cancer. Front. Genet..

[81] Verheyen E.M. (2022). The power of Drosophila in modeling human disease mechanisms. Dis. Model. Mech..

[82] Ingham P.W. (2018). From Drosophila segmentation to human cancer therapy. Development.

[83] Ashton-Beaucage D., Therrien M. (2017). How Genetics Has Helped Piece Together the MAPK Signaling Pathway. Methods Mol. Biol..

[84] Bejsovec A. (2018). Wingless Signaling: A Genetic Journey from Morphogenesis to Metastasis. Genetics.

[85] Salazar J.L., Yamamoto S. (2018). Integration of Drosophila and Human Genetics to Understand Notch Signaling Related Diseases. Adv. Exp. Med. Biol..

[86] Nusse R., Clevers H. (2017). Wnt/beta-Catenin Signaling, Disease, and Emerging Therapeutic Modalities. Cell.

[87] Zhang C., Brunt L., Ono Y., Rogers S., Scholpp S. (2024). Cytoneme-mediated transport of active Wnt5b-Ror2 complexes in zebrafish. Nature.

[88] Mattes B., Dang Y., Greicius G., Kaufmann L.T., Prunsche B., Rosenbauer J., Stegmaier J., Mikut R., Ozbek S., Nienhaus G.U. (2018). Wnt/PCP controls spreading of Wnt/beta-catenin signals by cytonemes in vertebrates. eLife.

[89] Rogers S., Zhang C., Anagnostidis V., Liddle C., Fishel M.L., Gielen F., Scholpp S. (2023). Cancer-associated fibroblasts influence Wnt/PCP signaling in gastric cancer cells by cytoneme-based dissemination of ROR2. Proc. Natl. Acad. Sci. USA.

[90] Hall E.T., Dillard M.E., Cleverdon E.R., Zhang Y., Daly C.A., Ansari S.S., Wakefield R., Stewart D.P., Pruett-Miller S.M., Lavado A. (2024). Cytoneme signaling provides essential contributions to mammalian tissue patterning. Cell.

[91] Wang Y., Nguyen T., He Q., Has O., Forouzesh K., Eom D.S. (2025). Cytoneme-mediated intercellular signaling in keratinocytes is essential for epidermal remodeling in zebrafish. eLife.

[92] Eom D.S., Parichy D.M. (2017). A macrophage relay for long-distance signaling during postembryonic tissue remodeling. Science.

[93] Lin X., Zhou Q., Zhao C., Lin G., Xu J., Wen Z. (2019). An Ectoderm-Derived Myeloid-like Cell Population Functions as Antigen Transporters for Langerhans Cells in Zebrafish Epidermis. Dev. Cell.

[94] Rustom A., Saffrich R., Markovic I., Walther P., Gerdes H.H. (2004). Nanotubular highways for intercellular organelle transport. Science.

[95] Koyanagi M., Brandes R.P., Haendeler J., Zeiher A.M., Dimmeler S. (2005). Cell-to-cell connection of endothelial progenitor cells with cardiac myocytes by nanotubes: A novel mechanism for cell fate changes?. Circ. Res..

[96] Boyineni J., Wood J.M., Ravindra A., Boley E., Donohue S.E., Soares M.B., Malchenko S. (2024). Prospective Approach to Deciphering the Impact of Intercellular Mitochondrial Transfer from Human Neural Stem Cells and Brain Tumor-Initiating Cells to Neighboring Astrocytes. Cells.

[97] Matejka N., Reindl J. (2020). Influence of alpha-Particle Radiation on Intercellular Communication Networks of Tunneling Nanotubes in U87 Glioblastoma Cells. Front. Oncol..

[98] Valdebenito S., Malik S., Luu R., Loudig O., Mitchell M., Okafo G., Bhat K., Prideaux B., Eugenin E.A. (2021). Tunneling nanotubes, TNT, communicate glioblastoma with surrounding non-tumor astrocytes to adapt them to hypoxic and metabolic tumor conditions. Sci. Rep..

[99] Korenkova O., Liu S., Prlesi I., Pepe A., Albadri S., Del Bene F., Zurzolo C. (2025). Tunneling nanotubes enable intercellular transfer in zebrafish embryos. Dev. Cell.

[100] Borcherding N., Brestoff J.R. (2023). The power and potential of mitochondria transfer. Nature.

[101] Guerra F., Arbini A.A., Moro L. (2017). Mitochondria and cancer chemoresistance. Biochim. Biophys. Acta (BBA)-Bioenerg..

[102] Ahmad T., Mukherjee S., Pattnaik B., Kumar M., Singh S., Kumar M., Rehman R., Tiwari B.K., Jha K.A., Barhanpurkar A.P. (2014). Miro1 regulates intercellular mitochondrial transport & enhances mesenchymal stem cell rescue efficacy. EMBO J..

[103] Venkataratnam Y., Mukherjee V., Rai N., Mahalingaswamy M., Shandilya D., Konar S., Nanjegowda N.B., Waghmare G., Nanjaiah N.D. (2025). Tunneling nanotubes between glioblastoma cells and T cells and hijack of mitochondria. J. Neuro-Oncol..

[104] Ravi V.M., Neidert N., Will P., Joseph K., Maier J.P., Kuckelhaus J., Vollmer L., Goeldner J.M., Behringer S.P., Scherer F. (2022). T-cell dysfunction in the glioblastoma microenvironment is mediated by myeloid cells releasing interleukin-10. Nat. Commun..

[105] Lu J., Zheng X., Li F., Yu Y., Chen Z., Liu Z., Wang Z., Xu H., Yang W. (2017). Tunneling nanotubes promote intercellular mitochondria transfer followed by increased invasiveness in bladder cancer cells. Oncotarget.

[106] Giannattasio S., Guaragnella N., Arbini A.A., Moro L. (2013). Stress-related mitochondrial components and mitochondrial genome as targets of anticancer therapy. Chem. Biol. Drug Des..

[107] Guerra F., Guaragnella N., Arbini A.A., Bucci C., Giannattasio S., Moro L. (2017). Mitochondrial Dysfunction: A Novel Potential Driver of Epithelial-to-Mesenchymal Transition in Cancer. Front. Oncol..

[108] Novak J., Nahacka Z., Oliveira G.L., Brisudova P., Dubisova M., Dvorakova S., Miklovicova S., Dalecka M., Puttrich V., Grycova L. (2025). The adaptor protein Miro1 modulates horizontal transfer of mitochondria in mouse melanoma models. Cell Rep..

[109] Ikeda H., Kawase K., Nishi T., Watanabe T., Takenaga K., Inozume T., Ishino T., Aki S., Lin J., Kawashima S. (2025). Immune evasion through mitochondrial transfer in the tumour microenvironment. Nature.

[110] Wang J., Liu X., Qiu Y., Shi Y., Cai J., Wang B., Wei X., Ke Q., Sui X., Wang Y. (2018). Cell adhesion-mediated mitochondria transfer contributes to mesenchymal stem cell-induced chemoresistance on T cell acute lymphoblastic leukemia cells. J. Hematol. Oncol..

[111] Sowinski S., Jolly C., Berninghausen O., Purbhoo M.A., Chauveau A., Kohler K., Oddos S., Eissmann P., Brodsky F.M., Hopkins C. (2008). Membrane nanotubes physically connect T cells over long distances presenting a novel route for HIV-1 transmission. Nat. Cell Biol..

[112] Eugenin E.A., Gaskill P.J., Berman J.W. (2009). Tunneling nanotubes (TNT) are induced by HIV-infection of macrophages: A potential mechanism for intercellular HIV trafficking. Cell. Immunol..

[113] Lv W., Li Z., Wang S., He J., Zhang L. (2024). A role for tunneling nanotubes in virus spread. Front. Microbiol..

[114] Van Prooyen N., Gold H., Andresen V., Schwartz O., Jones K., Ruscetti F., Lockett S., Gudla P., Venzon D., Franchini G. (2010). Human T-cell leukemia virus type 1 p8 protein increases cellular conduits and virus transmission. Proc. Natl. Acad. Sci. USA.

[115] Roberts K.L., Manicassamy B., Lamb R.A. (2015). Influenza A virus uses intercellular connections to spread to neighboring cells. J. Virol..

[116] Pepe A., Pietropaoli S., Vos M., Barba-Spaeth G., Zurzolo C. (2022). Tunneling nanotubes provide a route for SARS-CoV-2 spreading. Sci. Adv..

[117] Rubio-Casillas A., Redwan E.M., Uversky V.N. (2022). SARS-CoV-2: A Master of Immune Evasion. Biomedicines.

[118] Djurkovic M.A., Leavitt C.G., Arnett E., Kriachun V., Martinez-Sobrido L., Titone R., Sherwood L.J., Hayhurst A., Schlesinger L.S., Shtanko O. (2023). Ebola Virus Uses Tunneling Nanotubes as an Alternate Route of Dissemination. J. Infect. Dis..

[119] Michita R.T., Tran L.B., Bark S.J., Kumar D., Toner S.A., Jose J., Mysorekar I.U., Narayanan A. (2025). Zika virus NS1 drives tunneling nanotube formation for mitochondrial transfer and stealth transmission in trophoblasts. Nat. Commun..

[120] Wang X., Bukoreshtliev N.V., Gerdes H.H. (2012). Developing neurons form transient nanotubes facilitating electrical coupling and calcium signaling with distant astrocytes. PLoS ONE.

[121] Ross C.A., Poirier M.A. (2004). Protein aggregation and neurodegenerative disease. Nat. Med..

[122] Bockman J.M., Kingsbury D.T., McKinley M.P., Bendheim P.E., Prusiner S.B. (1985). Creutzfeldt-Jakob disease prion proteins in human brains. N. Engl. J. Med..

[123] MacDonald M.E., Ambrose C.M., Duyao M.P., Myers R.H., Lin C., Srinidhi L., Barnes G., Taylor S.A., James M., Groot N. (1993). A novel gene containing a trinucleotide repeat that is expanded and unstable on Huntington’s disease chromosomes. Cell.

[124] Polymeropoulos M.H., Lavedan C., Leroy E., Ide S.E., Dehejia A., Dutra A., Pike B., Root H., Rubenstein J., Boyer R. (1997). Mutation in the alpha-synuclein gene identified in families with Parkinson’s disease. Science.

[125] Grundke-Iqbal I., Iqbal K., Tung Y.C., Quinlan M., Wisniewski H.M., Binder L.I. (1986). Abnormal phosphorylation of the microtubule-associated protein tau (tau) in Alzheimer cytoskeletal pathology. Proc. Natl. Acad. Sci. USA.

[126] Glenner G.G., Wong C.W. (1984). Alzheimer’s disease: Initial report of the purification and characterization of a novel cerebrovascular amyloid protein. Biochem. Biophys. Res. Commun..

[127] Victoria G.S., Zurzolo C. (2017). The spread of prion-like proteins by lysosomes and tunneling nanotubes: Implications for neurodegenerative diseases. J. Cell Biol..

[128] Zhou C., Huang M., Wang S., Chu S., Zhang Z., Chen N. (2024). Tunneling nanotubes: The transport highway for astrocyte-neuron communication in the central nervous system. Brain Res. Bull..

[129] Chakraborty R., Belian S., Zurzolo C. (2023). Hijacking intercellular trafficking for the spread of protein aggregates in neurodegenerative diseases: A focus on tunneling nanotubes (TNTs). Extracell. Vesicles Circ. Nucl. Acids.

[130] Gousset K., Schiff E., Langevin C., Marijanovic Z., Caputo A., Browman D.T., Chenouard N., de Chaumont F., Martino A., Enninga J. (2009). Prions hijack tunnelling nanotubes for intercellular spread. Nat. Cell Biol..

[131] Victoria G.S., Arkhipenko A., Zhu S., Syan S., Zurzolo C. (2016). Astrocyte-to-neuron intercellular prion transfer is mediated by cell-cell contact. Sci. Rep..

[132] Chastagner P., Loria F., Vargas J.Y., Tois J., IDiamond M., Okafo G., Brou C., Zurzolo C. (2020). Fate and propagation of endogenously formed Tau aggregates in neuronal cells. EMBO Mol. Med..

[133] Chakraborty R., Nonaka T., Hasegawa M., Zurzolo C. (2023). Tunnelling nanotubes between neuronal and microglial cells allow bi-directional transfer of alpha-Synuclein and mitochondria. Cell Death Dis..

[134] Chakraborty R., Palese F., Samella P., Testa V., Montero-Muñoz J., Syan S., Nonaka T., Hasegawa M., Consiglio A., Zurzolo C. (2026). Impaired alpha-Synuclein aggregate clearance in neuronal cells drive their spread to microglia through tunneling nanotubes. Nat. Commun..

[135] Herrmann J.M. (2024). Mitochondria: The beating heart of the eukaryotic cell. FEBS Open Bio.

